# Supplementary material for: Prospects for a prolonged slowdown in global warming in the early 21st century
Source: Nat Commun. 2016 Nov 30;7:13676. doi: 10.1038/ncomms13676 (PMC5141387; doi:10.1038/ncomms13676)
Supplement: Supplementary Information — Supplementary Figures 1-5, Supplementary Notes 1-3 and Supplementary References 1-2 [file ncomms13676-s1.pdf]

Supplementary Information

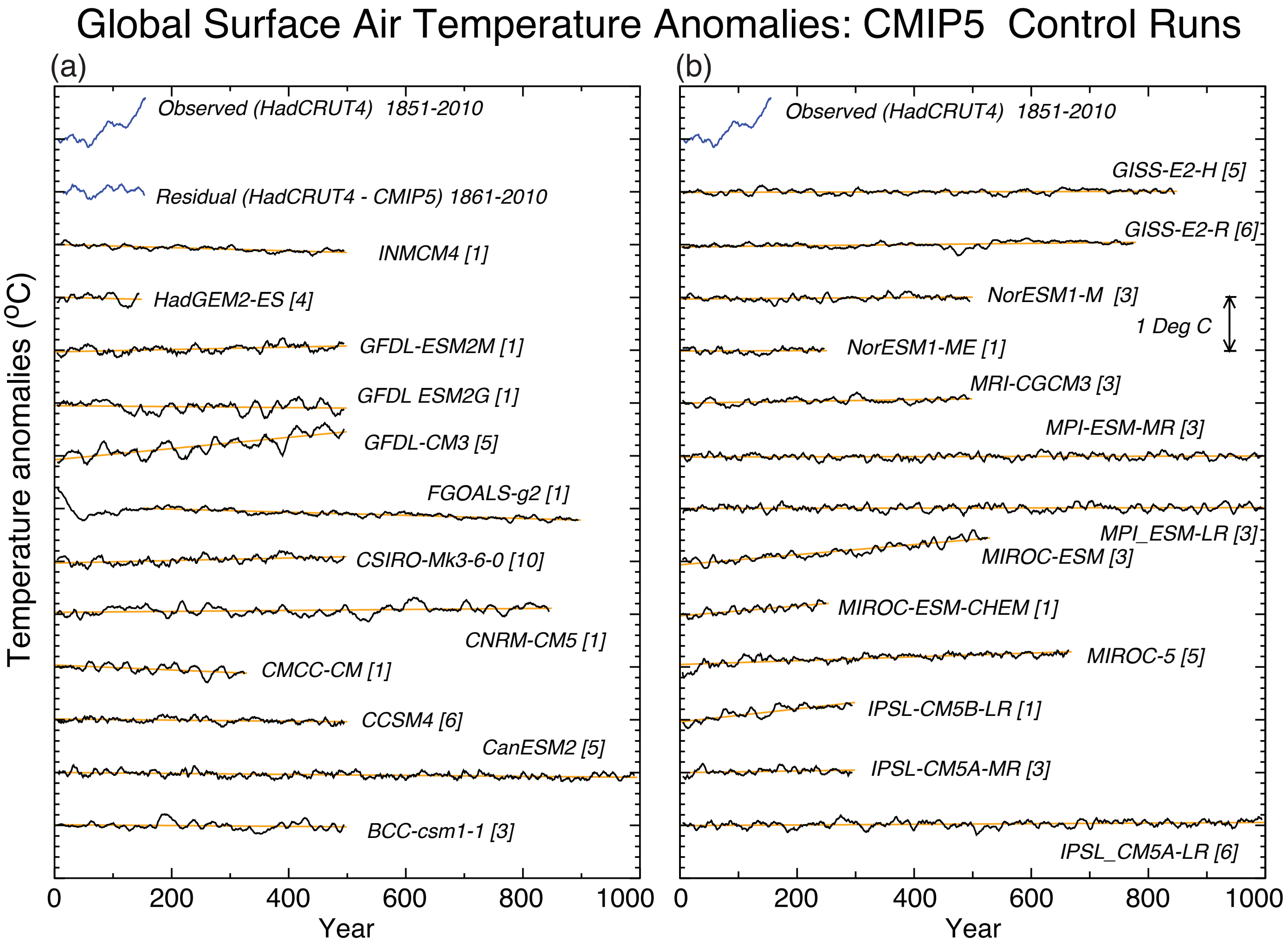

Supplementary Figure 1. Global mean surface air temperature anomalies from CMIP5 control runs and observations. Black lines are 10-yr running means from 25 CMIP5 control runs, with orange lines depicting long-term linear trends (drift). See labels for identification of individual models. Numbers in brackets provide the number of All-Forcing historical run ensemble members used for each model. Also shown (blue lines) are observed global mean temperature anomalies from HadCRUT4.4 and a residual estimate of observed internal variability obtained by subtracting the CMIP5 multimodel ensemble All-Forcing response from the HadCRUT4.4 data. The figure shows that CM3 generally simulates higher levels of internal multidecadal variability than the other CMIP5 models. See labels on plot for further information. A sample 1 degree Celsius anomaly reference length is shown in (b) by the vertical arrow scale.

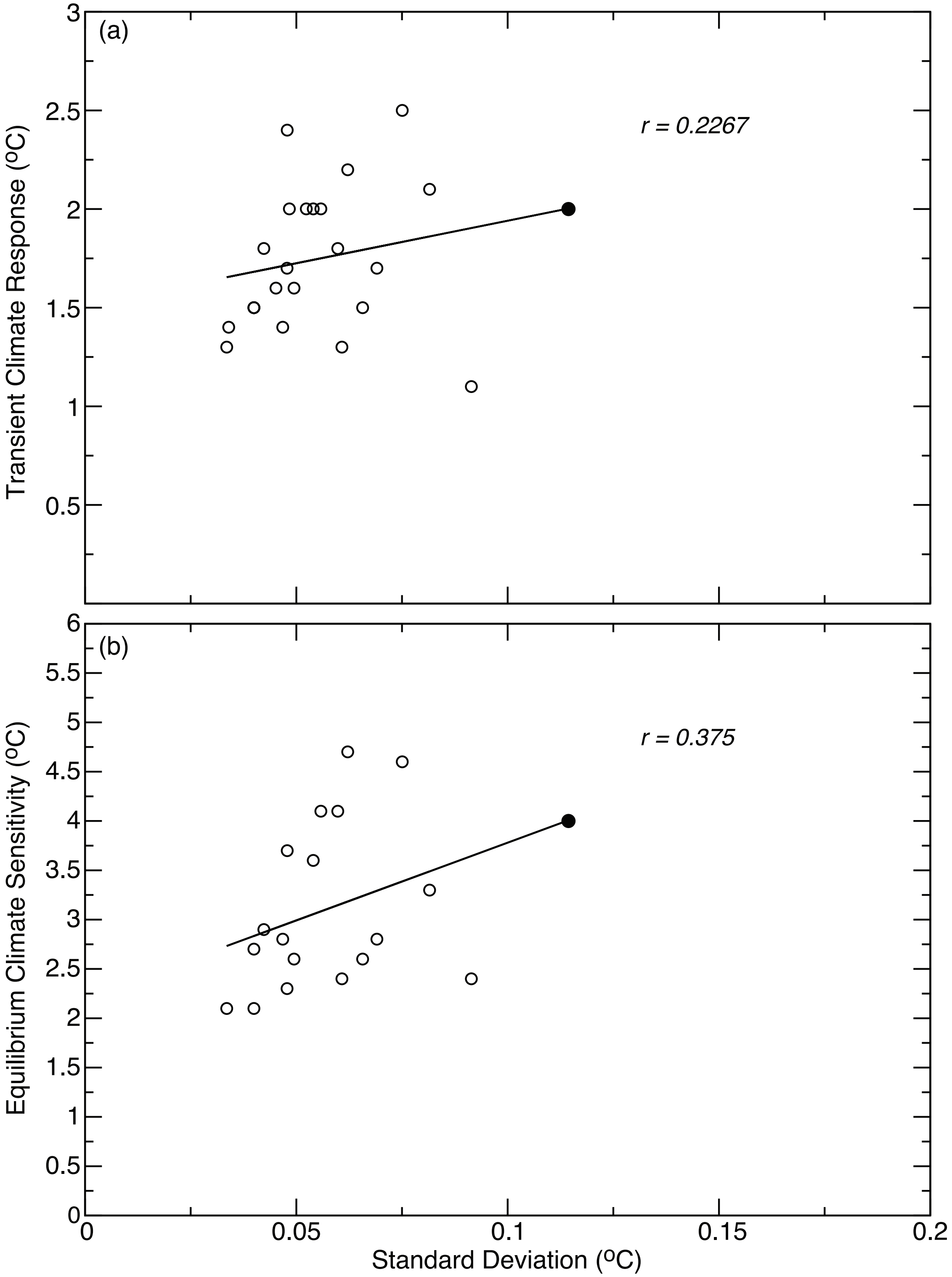

Supplementary Figure 2. Scatterplots of model climate sensitivity versus low-frequency internal variability for the CMIP5 models. Shown are the a) transient climate response (TCR) or b) equilibrium climate sensitivity (ECS) of the CMIP5 models vs. the standard deviation of 10-yr running mean global surface air temperature anomalies from the detrended long-term control runs shown in Supplementary Figure 1. The TCR and ECS values are those available from Table 9.5 of ref 1. Solid black dot: GFDL CM3 model. Solid line: linear trend, with correlation coefficients r.

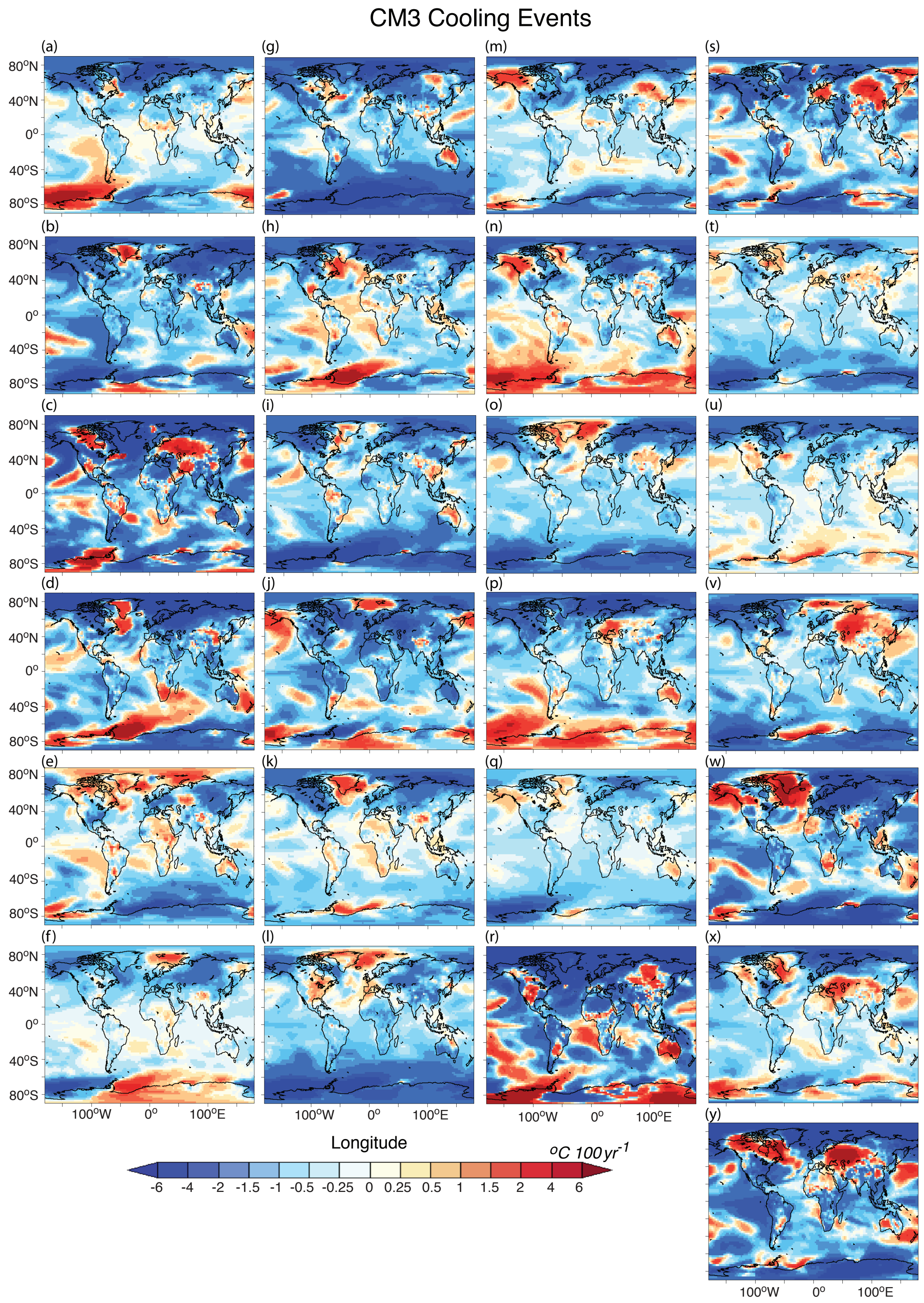

Supplementary Figure 3. Linear trend maps for 25 pronounced multidecadal cooling events from the CM3 control run. The segments (date ranges of the trends) are shown by the 25 line segments connecting pairs of circles in Fig. 2. Unit: $^{o}C\ 100\ yr^{-1}$.

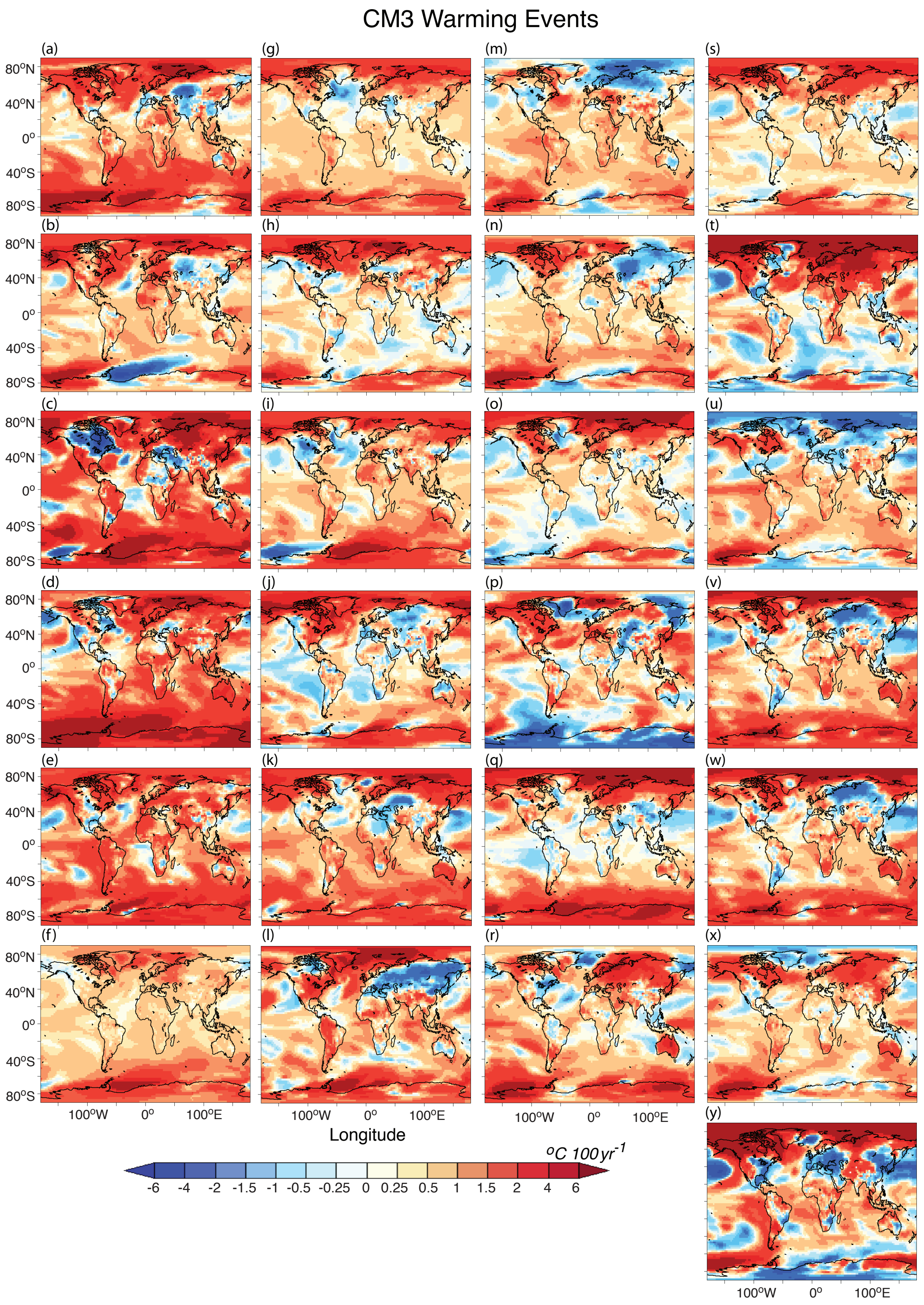

Supplementary Figure 4. Linear trend maps for 25 pronounced multidecadal warming events from the CM3 control run. Labeling and units as in Supplementary Figure 3. The segments used are not depicted in Fig. 2 as they are for the cooling events.

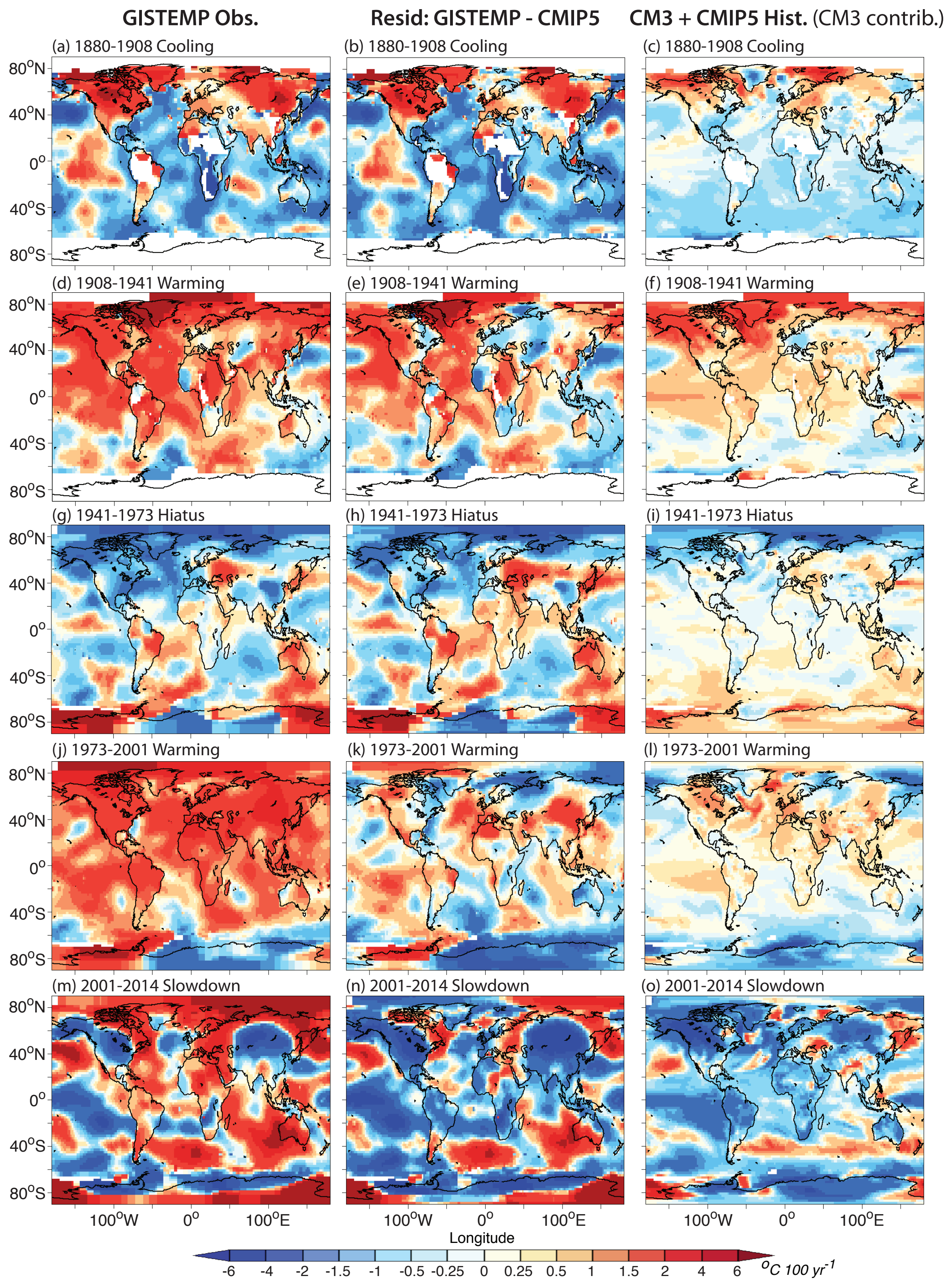

Supplementary Figure 5. Estimates of the internal variability contribution to observed trends. As in Fig. 6, but panels (b, e, h, k, and n) show estimates of the internal variability trend over each epoch, estimated as the observed trend minus the CMIP5 All-Forcing ensemble mean trend for that epoch. For panels (c, f, i, l, and o), a second internal variability estimate is shown, computed as for Fig. 6 panels (c, f, i, l, and o) but with the All-Forcing contribution to the trend subtracted. The first estimate does not consider internal variability simulations in estimating the contribution of internal variability to observed trends, while the second estimate incorporates CM3's internal variability in estimating the internal variability contribution to observed trends. .

Supplementary Notes.

Supplementary Note 1. Internal variability compared to climate sensitivity. The CM3 model is relatively sensitive to radiative forcing perturbations, with a transient climate response (TCR) of 2.0^{o}C and equilibrium climate sensitivity (ECS) to CO_2 doubling[1] of 4.0^{o}C, with an updated estimate[2] of 4.6 ^{o}C. A question arises whether CM3's relatively high climate sensitivity implies that it may have excessive internal climate variability. To explore this, we compare the CMIP5 model TCR and ECS values against the standard deviation of 10-year running mean global mean surface air temperature from their pre-industrial control runs (Supplementary Figure 2). The results show only a weak inter-model relationship between climate sensitivity and internal multidecadal variability of global mean temperature, with a correlation across the models of 0.23 for TCR and 0.38 for ECS. The analysis does not provide a strong basis for concluding that the CM3 model has excessive internal climate variability.

Supplementary Note 2. Variability of Internal Events in CM3. The 25 large cooling and warming events identified in the CM3 control run (Fig. 2) are further explored here. Composite surface air temperature trend maps for the 25 large global warming (or 25 cooling) events were shown in Fig. 4. The composite maps show that during strong warming or cooling events, the model's surface temperature anomalies have enhanced amplitude in the extratropics and high latitudes of both hemispheres. While the tropical and subtropical Pacific anomalies in the

composite maps are of the same sign as the global mean anomalies, they are smaller in magnitude than the high-latitude/extratropical anomalies.

To show how the characteristics of the warming and cooling events vary from event to event, the trend maps for the 25 individual multidecadal cooling and 25 warming episodes from the CM3 control run are shown in Supplementary Figures 3 and 4, respectively. These show that the strongest anomalies for individual warming or cooling multidecadal events tend to occur in the higher latitudes of one or both hemispheres. Some of the events have a strong eastern tropical Pacific signature, but this is not clearly present in many of the events, and eastern tropical Pacific anomalies can even be of opposite sign to the near-global mean anomaly in some episodes. Widely varying spatial structures are shown, with typically a relatively large high-latitude expression in either one or both hemispheres. Assuming these model-based results are applicable to the real world, they would suggest that different patterns or flavors of internal multidecadal variability may be applicable, depending on the event in question.

Supplementary Note 3. Alternative internal variability contribution estimates. Alternative estimates of the contribution of internal variability to the observed trend patterns during each of the five 20th century global mean temperature are shown in Supplementary Figure 5. The first set of estimates (panels b, e, h, k, n) is computed as the observed trend minus the CMIP5 All-Forcing ensemble mean trend for that epoch. An alternative estimate of the internal variability contribution (Supplementary Figure 5, panels c, f, i, l, o) is obtained by taking the trend maps combining CMIP5 All-Forcing ensemble and CM3 internal variability that fit the observations relatively well (Fig. 6, panels c, f, i, l, o), and subtracting from these the CMIP5 All-Forcing ensemble mean response. The resulting residual is the set of estimates shown in Supplementary

Figure 5 (panels c, f, i, l, o). Note that the first estimation method does not include the CM3 control run in computing an estimate, while the second method does include CM3 simulated variability. These two sets of internal variability contribution estimates show that while CM3 internal variability can help explain some aspects of the observed trends, there can still be notable differences between various estimates of the internal variability contribution to observed trends based on details of the method used to construct the estimates.

Supplementary Material References

1. Flato, G., Marotzke, J., Abiodun, B., Braconnot, P., Chou, S. C., Collins, W., Cox, P., Driouech, F., Emori, S., Eyring, V., Forest, C., Gleckler, P., Guilyardi, E., Jakob, C., Kattsov, V., Reason, C. & Rummukainen, M. Evaluation of Climate Models. In: *Climate Change 2013: The Physical Science Basis. Contribution of Working Group I to the Fifth Assessment Report of the Intergovernmental Panel on Climate Change* [Stocker, T.F., Qin, D., Plattner, G.-K., Tignor, M., Allen, S. K., Boschung, J., Nauels, A., Xia, Y., Bex, V. & Midgley, P. M. (eds.)]. Cambridge University Press, Cambridge, United Kingdom and New York, NY, USA (2013).

2. Winton, M., Adcroft, A., Griffies, S. M., Hallberg, R. W., Horowitz, L. W., & Stouffer, R. J. Influence of ocean and atmosphere components on simulated climate sensitivities. *J. Climate*, **26**, 231–245, doi: 10.1175/JCLI-D-12-00121.1 (2013).
